# Photocatalytic and theoretical study of CoS nanoparticles for sustainable dye removal from wastewater

**DOI:** 10.1038/s41598-025-13932-1

**Published:** 2025-08-21

**Authors:** Heba M. El Sharkawy, Ghada E. Khedr, Esraa M. El-Fawal

**Affiliations:** Department of Analysis and Evaluation, Egyptian Petroleum Research Institute, Nasr City, Cairo, 11727 Egypt

**Keywords:** Photodegradation, Dyes, Water treatment, DFT, Pollution remediation, Photocatalysis, Density functional theory, Computational chemistry, Quantum chemistry

## Abstract

Photocatalytic degradation has emerged as a promising approach for addressing dye-laden wastewater from industrial effluents. In this study, a cost-effective cobalt sulfide (CoS) photocatalyst was synthesized via a simple precipitation method and employed for the visible-light-driven degradation of cationic methylene blue (MB) and anionic methyl red (MR) dyes. The as-prepared CoS was characterized using XRD, HR-TEM, FE-SEM, DRS, and PL techniques, revealing a hexagonal phase structure, uniform spherical morphology with particle sizes of 15–22 nm, a mesoporous surface with a BET-specific surface area of 33.6 m²·g⁻¹, and a narrow band gap of 1.6 eV. Under optimized conditions, CoS demonstrated excellent photocatalytic performance, achieving 97.7% degradation of MB and 75.3% degradation of MR within 90 min under visible light. Kinetic analysis showed a pseudo-first-order reaction with rate constants of 0.03 min⁻¹ for MB and 0.01 min⁻¹ for MR. Density functional theory (DFT) simulations further elucidated the adsorption configurations and energetics of both dyes on the CoS (100) surface, revealing stronger adsorption of MB compared to MR. These findings highlight the potential of CoS as an affordable and efficient photocatalyst for sustainable wastewater remediation applications.

## Introduction

The photocatalytic degradation of organic dyes has gained significant attention due to its promising applications in environmental remediation and wastewater treatment^1^. Among these dyes, methylene blue (MB) and methyl red (MR) are widely used in industries such as textiles, pharmaceuticals, and food processing. However, their release into aquatic ecosystems poses serious environmental and health risks because of their persistence, toxicity, and resistance to conventional treatment methods^2–7^.

In response to these challenges, the utilization of photocatalytic processes for the degradation of MR, MB and other organic pollutants has emerged as a promising approach^8–10^. Photocatalysis, employing semiconductor materials. Furthermore, semiconductor-based photocatalysis harnesses solar or artificial light to initiate oxidative reactions, leading to the breakdown of organic pollutants into non-toxic byproducts^11–17^. Hence, photocatalysis has emerged as a committed avenue for the degradation of organic pollutants, offering an environmentally friendly and cost-effective solution to mitigate water contamination. Semiconductors like zinc oxide (ZnO), titanium dioxide (TiO_2_), and other metal oxides have been extensively applied as a photocatalysis for decomposition of polluted water^18,19^. While metaloxide photocatalysts such as TiO₂, ZnO, SnO₂, and WO₃ are well-known for their chemical stability and nontoxicity, they suffer from significant limitations that hinder real-world application^20^. While TiO₂ and ZnO have been foundational in photocatalysis, their wide band gaps (~ 3.0–3.3 eV) restrict their activation to the UV region, which comprises only about 5% of the solar spectrum, and they exhibit high rates of electron–hole recombination—resulting in limited visible-light efficiency^21,22^. Moreover, emerging materials such as metal-organic frameworks (MOFs) and doped carbon-based nanomaterials, although promising, pose challenges related to complex synthesis, poor scalability, and high cost. Additionally, many oxide-based photocatalysts suffer from poor pollutant adsorption and difficult recovery from aqueous media, which reduces their sustainability.

Among the various semiconductor materials, cobalt sulfide (CoS) has garnered worthy concern due to its special properties such as favorable band structure, high surface area, and excellent photoactivity^23,24^rendering it suitable for applications in photocatalytic processes^25–28^. CoS stands out due to its narrow band gap (≈ 1.0–1.5 eV) enabling strong absorption in the visible-light region^29^. CoS also features a high specific surface area with abundant active sites, enhancing adsorption and facilitating rapid redox reactions for pollutant degradation. Furthermore, its tunable electronic structure and lower recombination rate of photogenerated electron–hole pairs have been demonstrated to significantly improve photocatalytic efficiency^30^. Compared to other metal sulfides (e.g., CdS, CuS), CoS offers advantages in terms of earth abundance, cost-effectiveness, and environmental benignity, while delivering equivalent or better performance in visible-light-driven dye removal^31,32^. These combined merits make CoS a compelling and sustainable choice for advancing dye-degrading wastewater treatment technologies. In contrast, sulfur-based semiconductor nanoparticles, such as CoS, provide several compelling advantages over oxide counterparts. They feature narrower band gaps that enable efficient visible-light absorption, improved charge separation, and enhanced adsorption of organic dyes through strong sulfur–pollutant interactions. Moreover, sulfur-based materials are composed of earth-abundant and environmentally benign components, and their hydrophilicity and surface properties can be easily tuned via heterostructuring or doping. These combined attributes make sulfurbased nanomaterials a rational and justified choice for advanced, sustainable photocatalytic wastewater treatment.

Methyl red (MR) and methylene blue (MB) are representative examples of synthetic organic dyes extensively utilized in textile, pharmaceutical, and dyeing industries. However, their discharge into water bodies poses serious environmental and health hazards, necessitating effective remediation^10^. In this context, the utilization of CoS as a photocatalyst for the degradation of MR and MB presents a promising approach towards the elimination of these recalcitrant pollutants.

Overall, this study endeavors to aid in the creation of effective and sustainable photocatalytic technologies for the removal of organic dyes from aqueous environments, thereby advancing environmental protection and water quality preservation.

While several CoS-based photocatalysts have been previously reported—such as CoS/CoO microspheres achieving ~ 98% degradation of methylene blue in 60 min via complex microsphere-membrane composites^33^ and CoS–gC₃N₄ heterojunctions employed for dye removal with multi-step hydrothermal synthesis^34^these studies predominantly target a single dye type (mostly cationic dyes) and offer limited mechanistic insight. Crucially, the simultaneous degradation of both cationic and anionic dyes remains underexplored^35^. Furthermore, there is a lack of molecular-level insight into dye adsorption and charge-transfer mechanisms on the CoS surface under visible-light irradiation. In this work, we address these gaps by employing both experimental photocatalytic tests and density functional theory simulations to investigate methylene blue (cationic) and methyl red (anionic) degradation. Our approach not only demonstrates efficient dual-dye removal but also elucidates the adsorption configurations, energetics, and reactive reactive species involved—providing a deeper mechanistic rationale and advancing CoS-based wastewater treatment technologies.

This work aims to design a cost-effective and efficient CoS photocatalyst for the visible-light-driven degradation of both cationic (methylene blue, MB) and anionic (methyl red, MR) dyes as model pollutants. The study integrates experimental approaches with density functional theory (DFT) calculations to gain deeper insights into the adsorption behavior and photodegradation mechanisms at the molecular level. The findings reveal that the synthesized CoS exhibits remarkable photocatalytic activity and good stability, underscoring its potential for practical applications in wastewater treatment. This dual experimental–theoretical approach provides a comprehensive understanding of CoS as a promising material and contributes to advancing the rational design of next-generation photocatalysts.

**Scheme 1:**
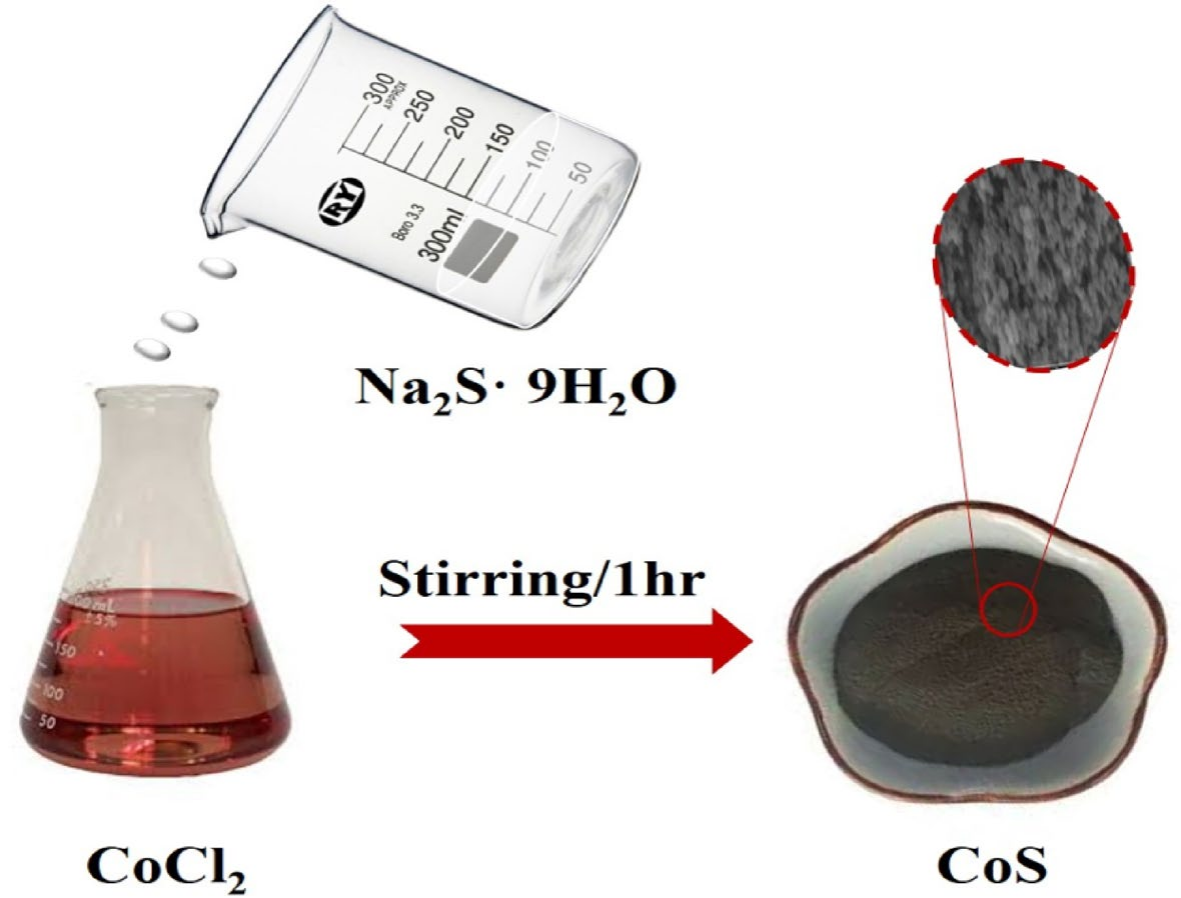
Schematic diagram illustrates the sequential preparation of the CoS nanopowder.

### Materials and characterization

Cobalt chloride (CoCl_2_), sodium sulphate (Na_2_S·9H_2_O), Methyl red (MR), and methylene blue (MB) were bought from Sigma-Aldrich. The remaining substances were all analytical reagent grade and were used without additional purification. Cu Kα radiation (Model X’Pert pro) was used to generate the X-ray powder diffraction (XRD) patterns on a powder X-ray diffractometer operating at 40 kV and 40 mA within the 2θ = 10–80^◦^ range. A high-resolution transmission electron microscope was used to characterize the microstructure morphologies (HRTEM, JEOL 2100, Japan). The steady state photoluminescence (PL) investigations were conducted at room temperature utilizing a fluorescence spectrophotometer (Perkin Elmer LS 55). DRS and scanning electron microscope (SEM).

### Preparation of cobalt sulfide

The following is how cobalt sulphide was prepared using a standard procedure: A magnetic stirrer was used to mix 100 ml of deionized water with 2.6 g of CoCl_2_. Next, in a different beaker, 2.8 g of Na_2_S· 9H_2_O was dissolved in 100 ml of deionized water. These quantities were chosen in order to maintain a molar ratio of 1 between cobalt and sulphur. After adding both solutions to a flask, we stirred for a full hour. After the reaction was finished, a precipitate was seen in the solutions. After centrifuging the solutions for 25 min at 1100 rpm and repeatedly washing them with 100% ethanol and distilled water, the product was allowed to dry at room temperature.

#### **I**nvestigation of photocatalytic activity

A 400 W halogen lamp was used as the light source in a photoreactor to study the photocatalytic degradation activity. Ten centimeters separate the dye solution from the halogen bulb. After adding 0.5 and 0.33 g/L of hetero-photocatalyst to 30 mL of a 20 ppm MB and MR dye solution, the mixture was agitated for 30 min in the dark to reach adsorption–desorption equilibrium. After 90 min after starting the photodegradation reaction, 5 mL of the suspension was taken out after 15 min. The UV-vis spectrophotometer was used to evaluate the obtained suspension.

### Computational methods

All calculations were carried out using the CASTEP code implemented in Materials Studio 2017, with the PBE functional and GGA^36–38^. Ultrasoft pseudopotentials were used with a cutoff energy of 400 eV and an energy convergence criterion of 10^−8^ and 0.01 eV/Å. A Monkhorst–Pack k-point grid of 3 × 3 × 1 was applied. The CoS structure was obtained from the Materials Project website, and the (100) surface was cleaved to simulate the experimental work. The plane was selected based on experimental XRD data. A vacuum slab of 25 Å was built to prevent interaction with the mirror image. The adsorption energy (E_ads_) was calculated using Eq. (1).

1 $$\:{E}_{ads}={E}_{(dye+surf)}-{E}_{dye}-{E}_{surf}$$

in which E_(dye+surf)_ is the combined surface and adsorbed dye energy, E(dye) is the relaxed dye energy, and E(surf) is the surface energy.

## Results and discussion

**Fig. 1:**
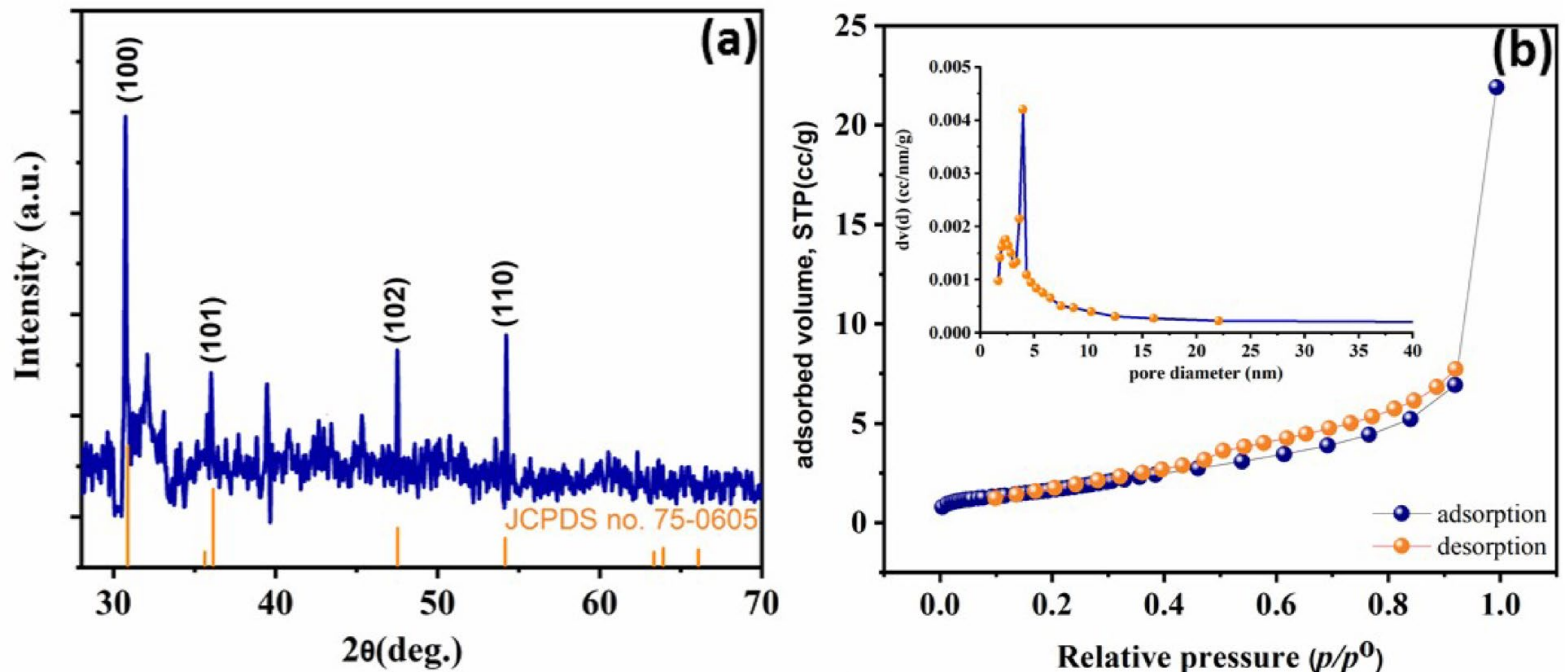
(**a**) XRD of CoS nanoparticle, (**b**) N_2_ adsorption/desorption isotherm of CoS nanoparticle, (inset: pore size distribution).

Scheme 1 represents the simple precipitation approach that was used to develop the CoS nanocomposite, which included mixing cobalt chloride and sodium sulphate at ambient temperature. The crystallinity and phase formed of the synthesized sample is demonstrated via XRD patterns of CoS^39,40^. It can be seen that the hexagonal phase (JCPDS no. 75–0605) of the CoS faces [100], [101], [102], and [110] are responsible for the peaks at 30.7, 36.0, 47.5, and 54.2, as shown in Fig. 1a.

The CoS textural characteristics was obtained via adsorption –desorption isotherm process. Essentially, the porosity distribution can be determined from the desorption branch of the isotherm generated by Barrett-Joyner-Halenda (BJH), and the specific surface area may be acquired using the Brunauer-Emmett-Teller (BET) method. The mesoporous material’s typical type IV isotherm with an H3 hysteresis loop was obvious in the as-prepared CoS nanoparticle. Additionally, as shown in Fig. 1b, the specific surface area of the nanoparticle assessed using the BET method was estimated to be 33.6 m^2^ g^−1^. This is provided by the majority of the pores, which are around 3.43 nm in size, and a small number of pores, which are 10.34 nm in size. These pores, all of which are mesopores, may act as conduits for the quick movement of ions resulting in complete contact of contaminants with the photocatalyst surface. Furthermore, the results of the BJH study indicate that the CoS nanoparticles have a pore volume of 0.036 cc/g.

**Fig. 2:**
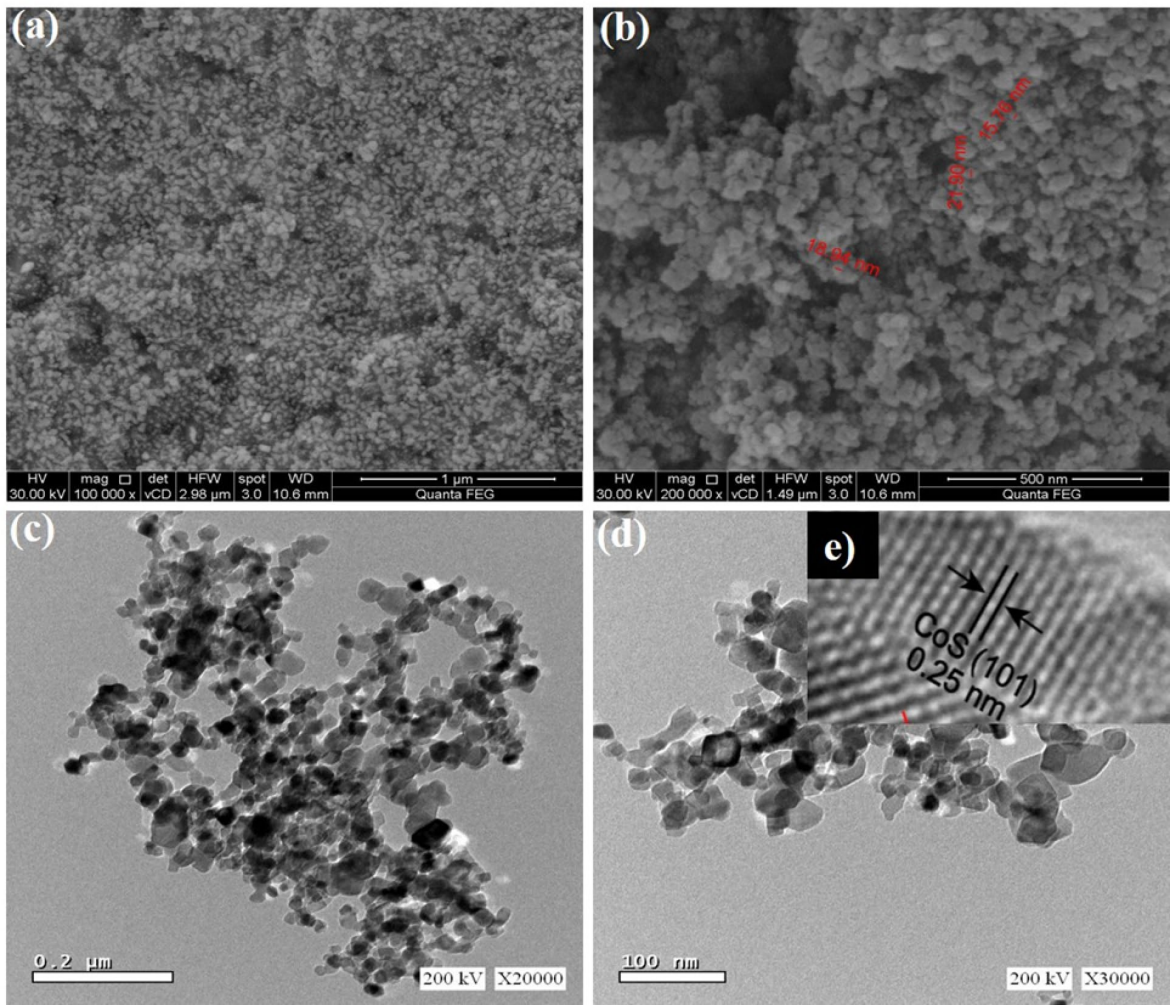
**a**, **b**)SEM of CoS nanoparticle and **c**, **d**) and inset **e**) HRTEM of CoS nanoparticle.

The surface morphology of the CoS photocatalyst with variety of magnification power was displayed by FESEM technique, Fig. 2a, b. The CoS was in a spherical morphology and the particles size was in the range of ~ 17–22 nm, thereby reduction of particle size increasing the specific surface area and adsorption of dyes. The surface areas would rise as a result of the higher porosities, which would then cause an increase in dye adsorptions and an improvement in the photocatalytic capabilities.

Close examination shows that, Fig. 2c, d, the TEM images for CoS display the consistent dispersion of CoS nanoparticles, which primarily have sizes between 15 and 22 nm. A comparatively large surface area could be provided by the nanoparticles’ well-distributed form. Moreover, demonstrates a distinct lattice spacing of 0.25 nm corresponding to the (101) plane of CoS^41^Fig. 2e.

**Fig. 3:**
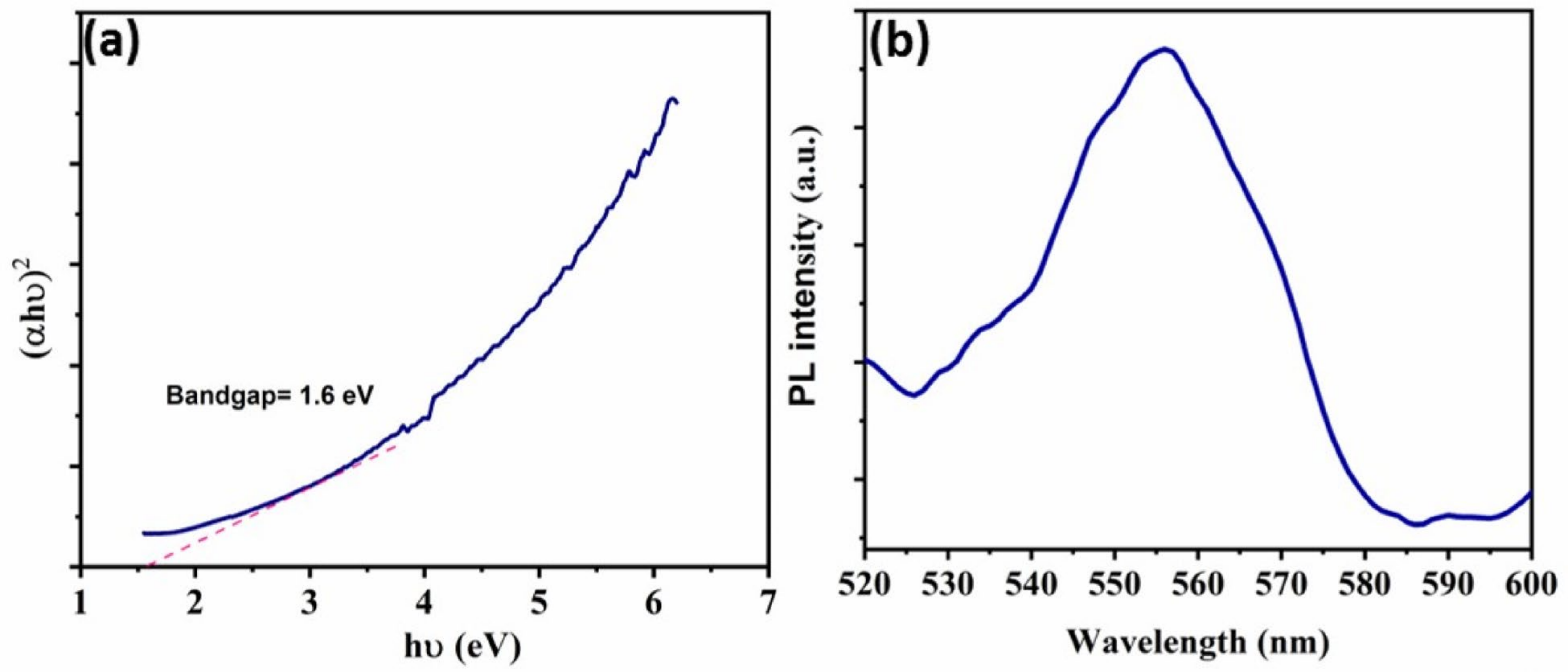
(**a**) Tauc plots of CoS and (**b**) Photoluminescence (PL) of CoS.

By extending the linear section of the (F(R) hυ)^2^ versus (hυ) curve towards the hυ axis, the band gap energy (Eg) was found. Equation (2) was applied to measure the optical band gap. The band gap energy was specified by plotting (αhυ)^2^ vs. (αυ), where α and hυ are the photon energy and absorption coefficient, respectively, A-constant, and n is the band gap (either 2 for direct transitions or 0.5 for indirect transitions)^42^.

2 $$\:{\left(\alpha\:h\upsilon\:\right)}^{n}=A(h\upsilon\:-{E}_{g})$$

According to Eq. (2), which is based on direct transitions, The band gap of CoS is 1.6 eV^39^., Fig. 3a. This low band gap makes the CoS of efficient visible light photocatalyst. From the perspective of photocatalysis, it is imperative that the photoinduced (e^−^)–(h^+^) couples be separated. Through the PL measurements, it could be possible to understand the attitude of these photo-generated charge carriers. A high recombination rate of photogenerated electrons and holes often points a high fluorescence intensity in PL spectra, however a low recombination rate can enhance photocatalytic efficacy. The PL spectra of the CoS-based nanocomposites by using excitation wave length of 350 nm (Fig. 3b) demonstrate a strong peak at 555 nm, which is linked with intrinsic band-to-band radiative recombination of moderately intense excited (e^−^) and (h^+^) recombination in the conduction and valence bands which is beneficial in photocatalytic efficiency by allowing an adequate charge carrier lifetime under exposure to visible light.

**Fig. 4:**
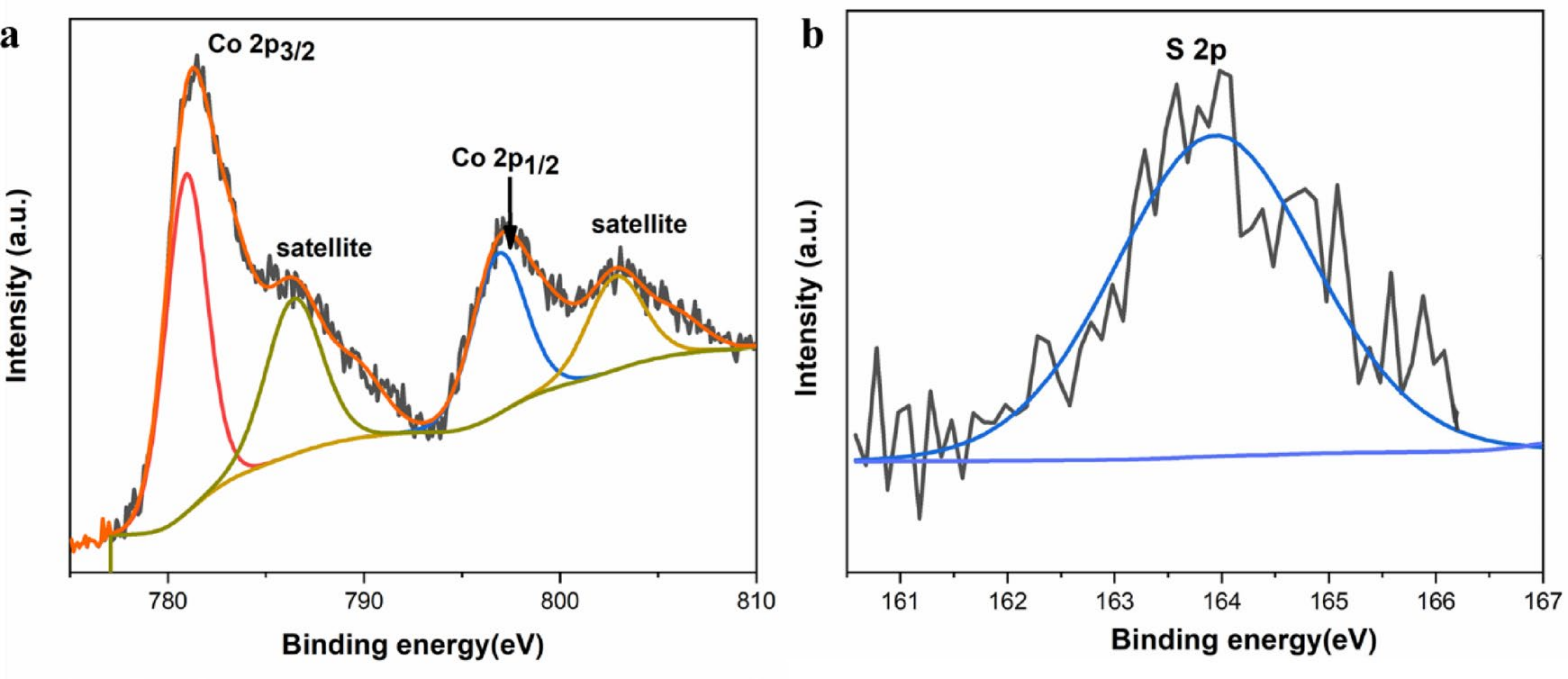
Deconvoluted XPS of CoS (**a**) Co (2p) spectra and (**b**) S (2p) spectra.

The elemental composition and oxidation states of the CoS nanospheres were investigated using X-ray photoelectron spectroscopy (XPS). As shown in Fig. 4a, the high-resolution Co 2p spectrum displays two main peaks at **781.0 eV** and **796.7 eV**, corresponding to **Co 2p₃/₂** and **Co 2p₁/₂**, respectively. Additionally, the satellite peaks at **785.7 eV** and **802.3 eV** further confirm the presence of **Co**^**2+**^ oxidation state. The high-resolution S 2p spectrum (Fig. 4b) the characteristic peak around **163.7 eV**, which are assigned to **S 2p₃/₂**, associated with Co–S bonding, and **S 2p₁/₂**, attributed to surface-bound divalent sulfur species^43^.

**Fig. 5:**
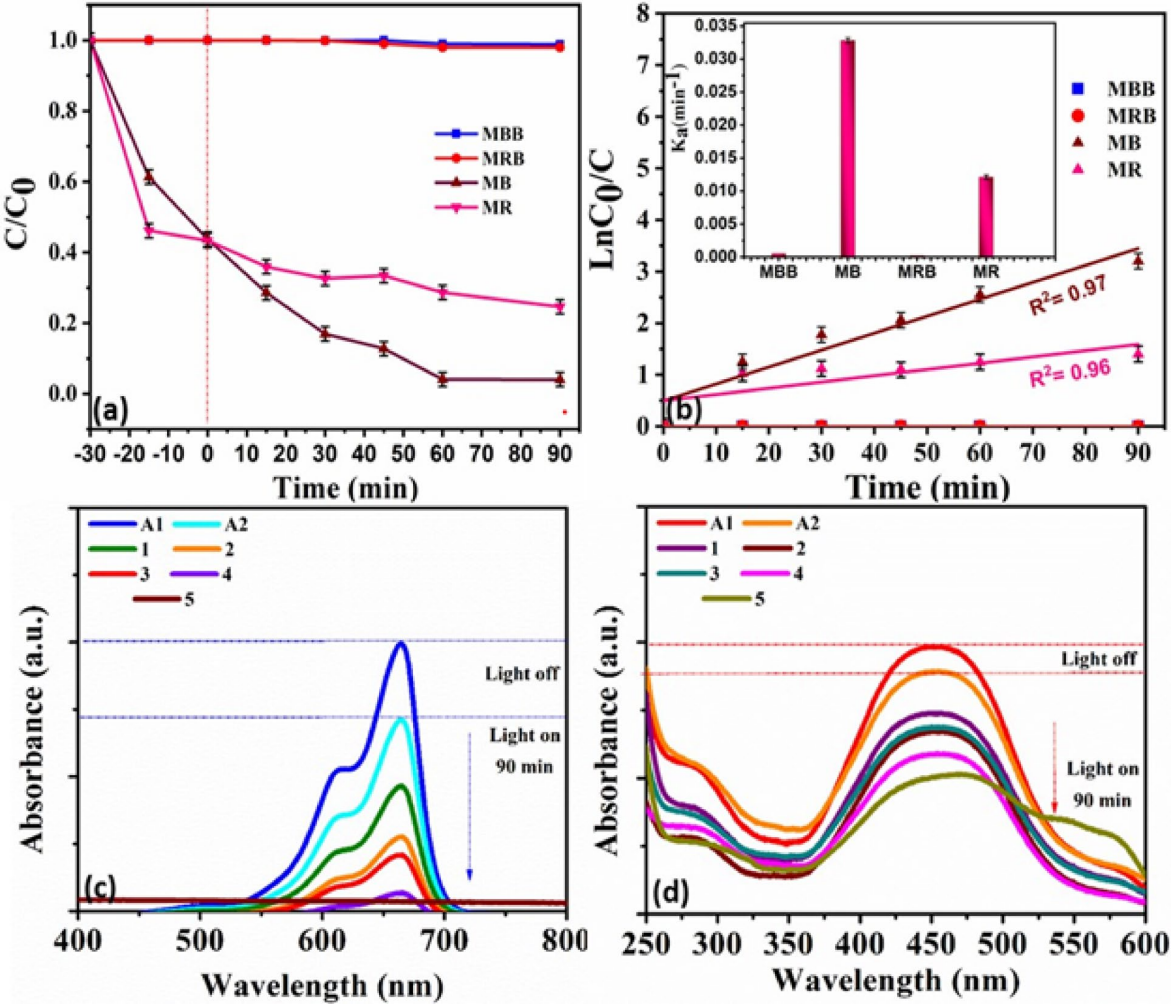
(**a**) photocatalytic degradation, (**b**) kinetic and inset apparent rate constants for the photocatalytic degradation of MB and MR by CoS nanocomposite, (**c**, **d**) UV–visible absorption of MB and MR dye solutions using CoS nanocomposite where A1,A2,referred to absorption of dye without illumination of light for 15 and 30 min and 1,2,3,4, and 5 referred to absorption of dye under visible light at irradiation times of 15, 30, 75, 60 and 90 min, respectively.

To explicate the photocatalytic performance of CoS, CoS as a photocatalyst for remediation of waste water, the photodegradation activity of CoS in cationic dye (MB) as well as anionic dye (MR) was scrutinized. Interestingly, the photocatalytic performance of CoS revealed its ability to act on cationic and anionic dyes, Fig. 5, suggesting promising photodegradation efficiency to degrade organic pollutant. Accordingly, the photocatalytic performance of CoS towards the cationic dye MB and the anionic dye MR was investigated separately in 20 ppm dye solution. Firstly, in order to evidence dye^’^s stability, photolysis test was established without addition of photocatalyst in darkness for 30 min followed by 90 min under exposure of light. Blank samples of MB and MR dyes, MB and MR were shown to be unsatisfactorily in the absence of photocatalyst, demonstrating the poor removal efficiency under photolysis^44,45^. Additionally, the photocatalytic degradation capability of CoS photocatalyst for MB and MR dyes was performed over a very small amount of CoS under visible light at irradiation times of 0, 30, 60, 90 min preceded with adsorption and desorption equilibrium. It is clear that the CoS was significantly better at degrading MB than MR during the visible light exposure. Where the achieved removal efficiency was 97.7% and 75.3% for MB and MR, respectively according to Eq. (3):

3 $$\:D\%=\left(\frac{\left({C}_{0}-C\right)}{{C}_{0}}\right)*100$$

where C_0_ is the initial concentration and C is the residual concentration of MB after the reaction.

In order to scrutinize the reason behind the observed enhanced performance of CoS towards degradation of cationic dye over that of the anionic one, the rate constant (ka) exhibited by cationic and anionic dye is estimated from the recorded dye concentration respect to its initial one at diverse photodegradation time based on the equation ln (C_0_/C) = k_a_ * t where k_a_ is the rate constant (min^−1^), C_0_ is the initial concentration (mg/L), and C is the reaction concentration of the dye solution when the irradiation time is t min., Fig. 5a. The cationic dye MB revealed a higher rate constant of 0.03 min^−1^, while the anionic dye MR exhibited a rate constant of only 0.01 min^−1^. This clearly indicates the photodegradation capability of the CoS composite to be utilized efficiently as a promising photocatalyst for the degradation of both MB and MR with a faster photodegradation rate for MB pollutant. Furthermore, the degradation process was pseudo-first-order based on the linear relationship obtained when plotting ln C_0_/C_t_ versus t^46,47^. In addition, as shown in Table 1, CoS displayed the highest photocatalytic efficiency toward both cationic and anionic pollutants under visible light irradiation in comparison with the photocatalytic activity of relevant nanocomposites.

**Table 1:** Comparison of photocatalytic efficiency of relevant photocatalysis with respect to the utilized CoS catalyst.

Photocatalyst	Dye type	Dye concentration (ppm)	Catalyst dose (g/L)	Time (min.)	Degradation (%)	Ref.
CoS_1.97_ (MG_1_T_1.5_)	MB	5	0.35	300	89.5%	^48^
CoS	MB	10	1	150	88%	^49^
g-C3N4@CdS	MB	5	1	170	97%	^50^
Ag_2_WO_4_-MoS_2_-GO (AgWMG)	MO	10	0.5	90	93.00	^51^
CdS NPs	MR	0.04 mM	1	135	77%	^52^
MoS_2_ NSs	MR	25	0.28	360	70.58%	^53^
CoS	**MB** **MR**	**20** **20**	**0.5** **0.33**	90	**97.7** **75.3**	**This work**

**Fig. 6:**
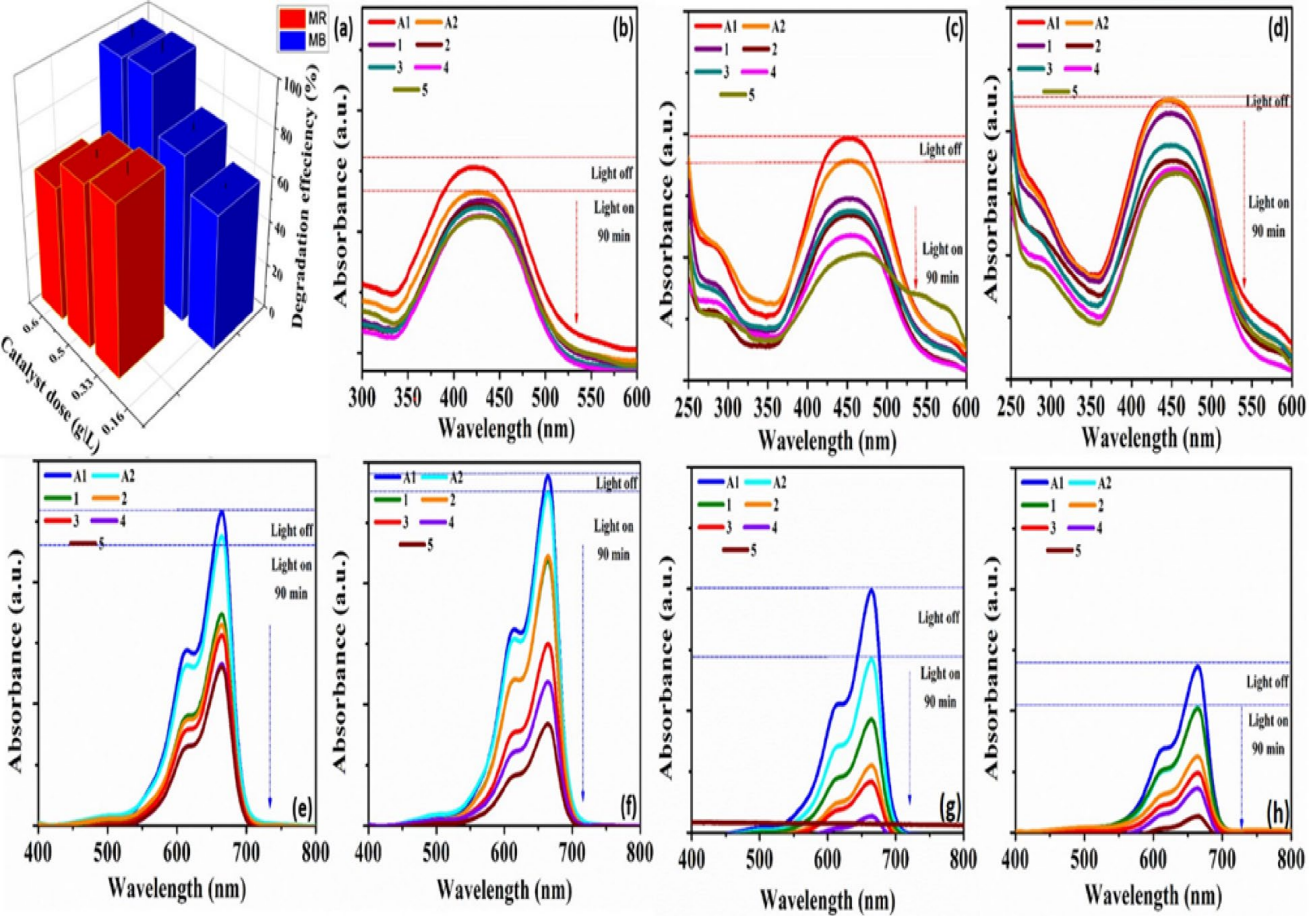
(**a**) dependency of photocatalytic degradation of MB and MR dye solutions on photocatalyst dosage and the corresponding UV–visible absorption of (**b**-**d**) MR dye and (**e**-**h**) of MB dye.

To better understand the enhanced photocatalytic activity of CoS towards both cationic (MB) and anionic (MR) dyes, zeta potential measurements were performed. Literature reports on similar cobalt sulfide nanomaterials indicate a moderately negative surface charge, e.g., approximately − 9.8 mV for CoS₂ nanoparticles at neutral pH, resulting in good colloidal stability and favorable electrostatic interactions^54^. A negative ζ-potential enhances the adsorption of positively charged dye molecules, such as MB, due to electrostatic attraction, while still allowing sufficient interaction with negatively charged dyes like MR through hydrogen bonding and van der Waals forces. This balanced surface charge, coupled with the mesoporous structure and visible-light absorption of CoS, contributes to its effective photocatalytic performance across different dye types.

Reasonable runs are conducted to find the ideal parameters for the photodegradation of anionic and cationic dyes. Selecting the optimal photocatalyst dosage is crucial in order to prevent overuse of the designed photocatalyst. Regarding this, Fig. 6 shows the influence of CoS dose on the photodegradation of the MB and MR dyes. We observed that, when the CoS dosage raised from 0.16 to 0.5 g/L, the MB degradation enhanced from 59.8 up to 97.7% and 75.3% (0.33 g/L) for the degradation MR dye. By raising the CoS photocatalyst concentration, the number of photoactive sites increases and the decomposition efficiency rises as a result. Additionally, the increased catalyst dose, provides a greater surface area for catalyst interaction and a higher concentration of free radicals per milliliter of the dye solution, consequently a higher dye removal efficiency is achieved^55^. Conversely, the degradation efficiency of MB dye decreased to 95.9% and MR dye to 59.6% when the dose was raised to 0.6 g/L, respectively. Low photoactive sites can arise from a lack of accessible radiation caused by an excess of catalyst in the solution, which also hinders visible light from reaching the reaction suspension^56^. Thus, 0.5 and 0.33 g/L were shown to be the optimal dosages for MB and MR photodegradation using CoS.

**Fig. 7:**
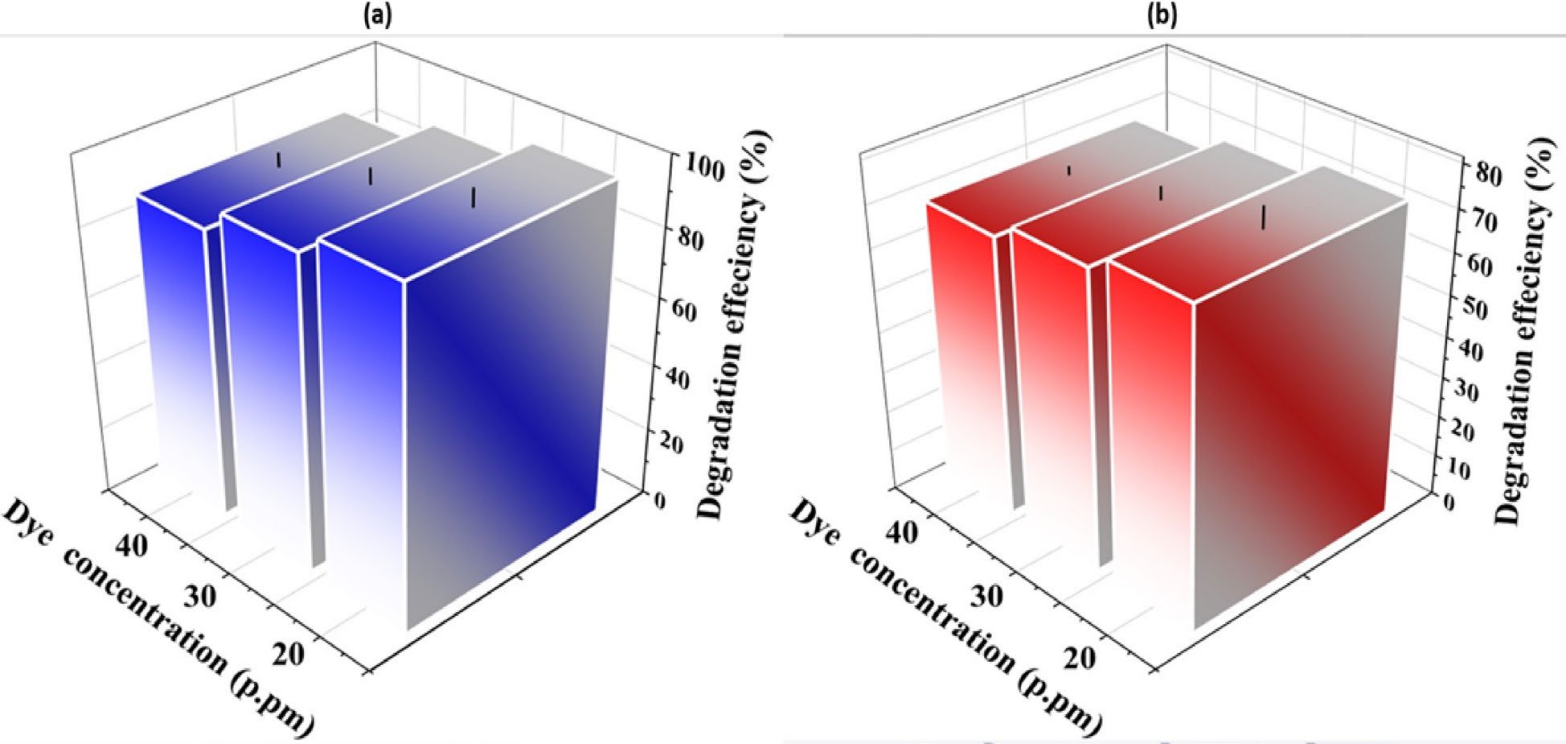
Dependency of photocatalytic degradation of MB and MR dye solutions on (**a**) MB (**b**) MR dyes concentrations.

The further important effective parameter is studying the influence of various dye concentrations of 20,30, and 40 ppm for photodegradation MB and MR under visible light irradiation with 0.5 and 0.33 g/L, the best performing dose as a photocatalyst dose for photodegradation MB and MR, respectively, Fig. 7. It is worth noting that the photodegradation results are associated with the pollutant concentration, as the efficiency of both MB and MR photodegradation reduces as the dye concentration rises. Under visible light irradiation for 90 min, a photodegradation of 97.7% is achieved by 20 ppm MB with 0.5 g/L of CoS photocatalyst. When the concentration is increased to 40 ppm, only 85.1% of the MB is photodegraded, Fig. 7a. Additionally, by increasing MR dye concentration, the same trend in dye degradation is observed. MR dye is exhibited photoremoval efficiency of 75.3% for 20 ppm and reached to 67.6% when its concentration was increased up to 40 ppm by 0.33 g/L of CoS, Fig. 7b. The reduction in the degradation efficiency is related to the inhibitory impact of the adsorbed dye as at high concentration, dye acts as a filter for incident light. Furthermore, increasing concentration of the dye would result in more light being absorbed, restricted the photon penetration to the photocatalyst surface on one side and lowered the number of active sites on photocatalyst surface on other side consequently, the photocatalytic degradation efficiency would be restricted.

**Fig. 8:**
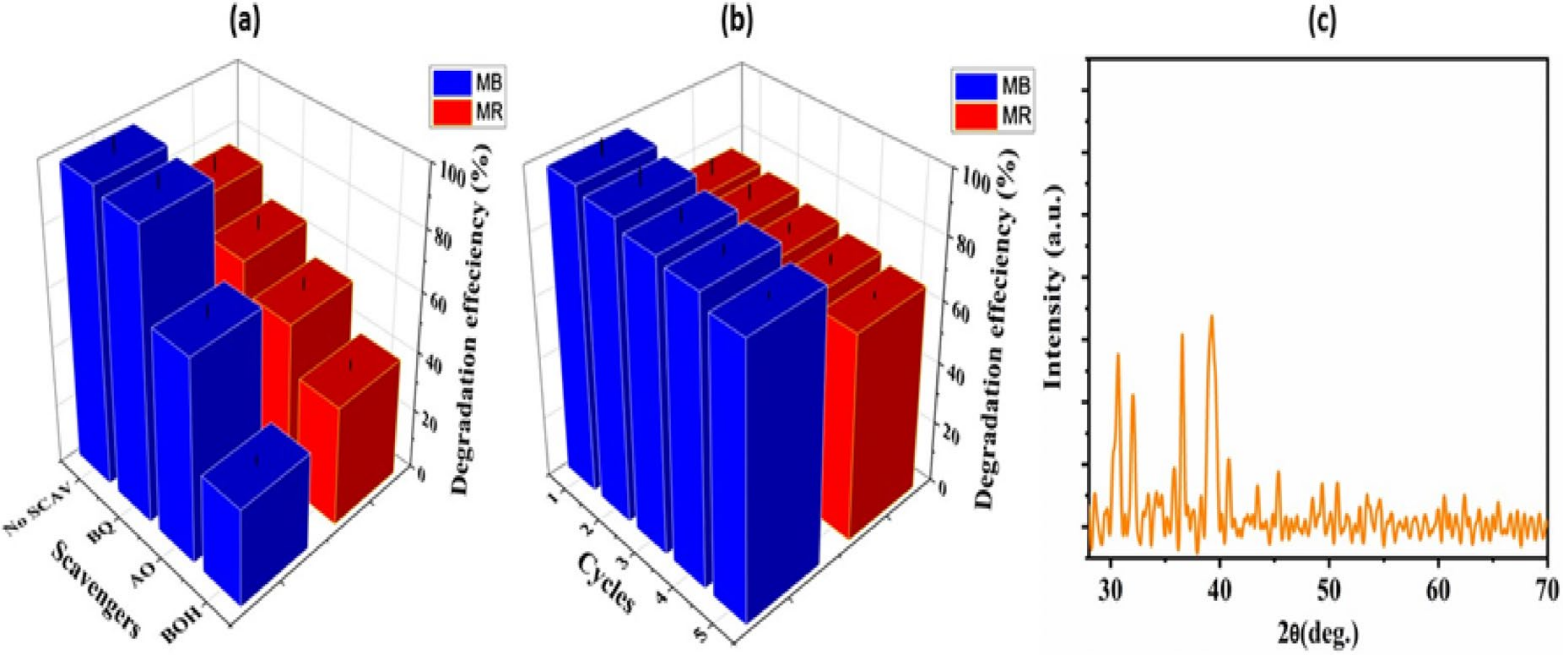
(**a**) MB and MR photocatalytic degradation by CoS composite with/without scavengers, (**b**) reusability of CoS and (**c**) the corresponding XRD spectra after photocatalytic degradation of MB and MR dye solutions.

The photogenerated charge carriers that emerge when visible light strikes the photocatalyst’s surface move from the valence band (VB) to the conduction band (CB) and leave holes in their path are key aspects during the photodegradation process. Once dye pollutants are exposed to photogenerated electron/hole pairs, they react and degrade them into environmentally benign CO_2_ and H_2_O, according to the following Eq. 

4 $$\:CS+h\upsilon\to\:{h}_{VB}^{+}+\:{e}_{CB}^{\_}$$

5 $$\:CS{e}_{CB}^{\_}+{O}_{2}\:\to\:CS+\:{O}_{2}^{\bullet\:-}$$

6 $$\:{O}_{2}+\:{H}_{2}O\to\:H{O}_{2}+{OH}^{-}$$

7 $$\:{HO}_{2}^{\bullet\:}+\:{H}_{2}O\to\:{OH}^{\bullet\:}+{H}_{2}{O}_{2}$$

8 $$\:{H}_{2}{O}_{2}\to\:2O{H}^{\bullet\:}$$

9 $$\:\:O{H}^{\bullet\:}+dye\to\:C{O}_{2}+{H}_{2}O$$

10 $$\:CS{h}_{VB}^{+}+\:dye\to\:C{O}_{2}+{H}_{2}O$$

Depending on the overall degradation mechanism, the investigation whether photo-generated reactive species are involved in the degradation of MB and MR dyes is illustrated via displaying the trapping experiments. Benzoquinone (BQ), ammonium oxalate (AO) and Butanol (BOH), were utilized in the trapping experiments as a scavenger for the reactive species of superoxide radical anions (•O_2_), photoinduced holes (h+) and hydroxyl radicals (•OH), respectively as depicted in Fig. 8a. It is observed that the by adding the scavengers the degradation efficiency of pollutant dyes were retarded compared to no scavengers. Furthermore, the trapping species are plumed the degradation efficiency to 95.7, 67.0, 32.5 and 65.3, 55.2, 39.8% in the presence of BQ, AO and BOH for photo decomposition of both MB and MR dyes, respectively. The aforementioned results suggest that the photocatalytic oxidation process depends critically on the •O_2_, h+, and •OH radicals^57,58^.

The supreme reuse property of the developed photocatalyst is significant for the efficient photocatalyst and implemented by successive cycling, Fig. 8b. The slight reduction of the photocatalytic oxidation efficiency may be due to the deactivation of the photocatalyst owing to the intermediate’s accumulation over the photocatalyst surface. Additionally, the XRD analysis of the fresh and recovered photocatalyst demonstrated no discernible changes towards its crystal structure indicating that its superior photocatalyst stability as shown in Fig. 8c.

**Fig. 9:**
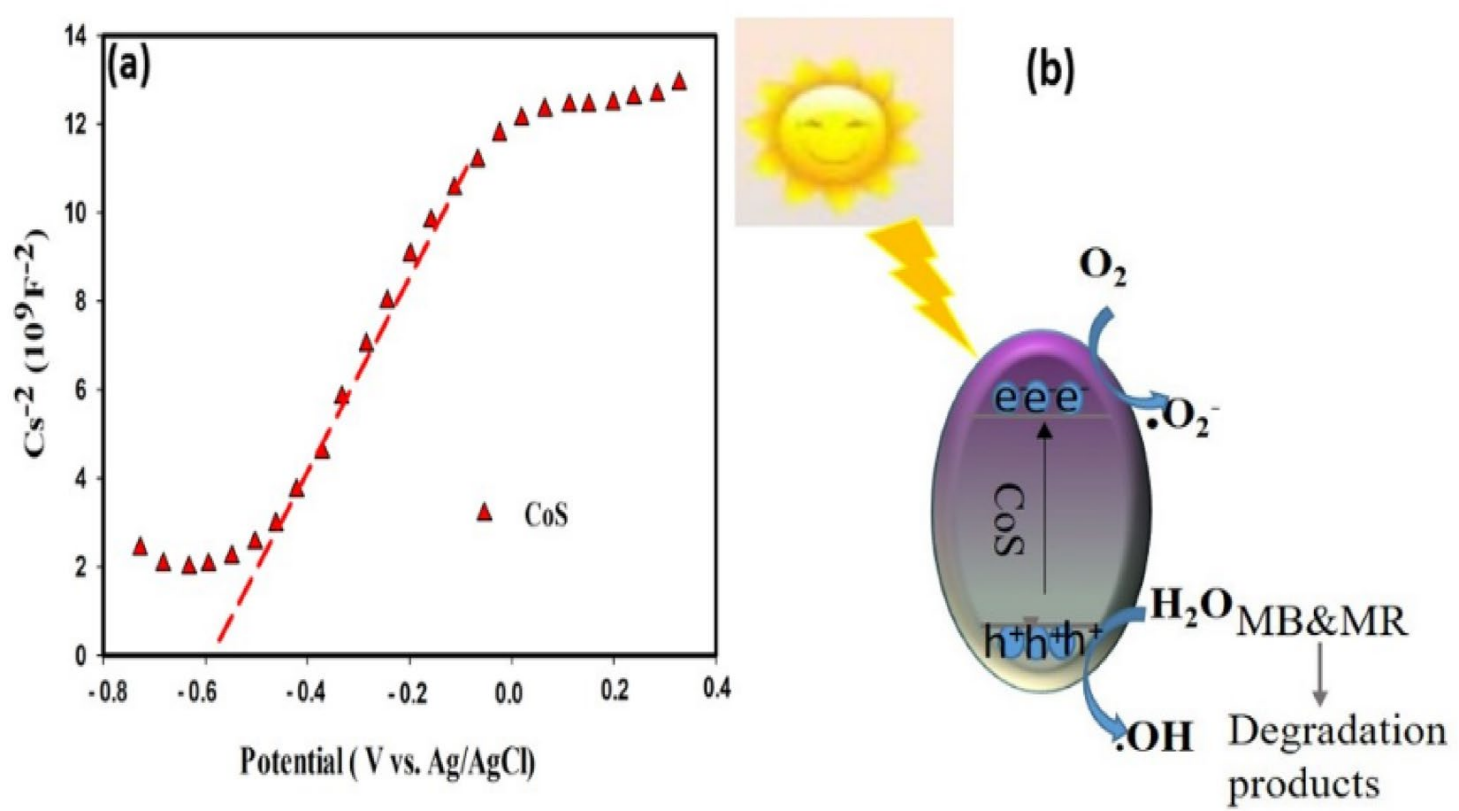
(**a**) Mott-Schottky analysis of CoS and (**b**) schematic diagram of mechanism of photocatalytic degradation.

To understand the electronic properties and photocatalytic activity of the synthesized CoS, a Mott-Schottky analysis was performed, as displayed in Fig. 9. The plot of Cs^−2^​ versus potential exhibits a positive slope, confirming the n-type semiconducting nature of the material. Based on an extrapolation of this data, the flat-band potential (V_fb_​) is determined to be −0.57 V vs. Ag/AgCl. Converting this to the Normal Hydrogen Electrode (NHE) scale (ENHE​=E_Ag/AgCl​_+0.197 V) places the V_fb_​ at approximately − 0.373 V vs. NHE, which serves as a close approximation for the conduction band minimum (E_CB_​). Given the optical band gap (E_g_​) of 1.6 eV, the valence band maximum (E_VB_​) is calculated to be approximately + 1.227 V vs. NHE. These band positions facilitate a powerful photocatalytic mechanism for the degradation of methylene blue and methyl red. Upon illumination, the photogenerated electrons in the conduction band (−0.373 V) are sufficiently energetic to reduce molecular oxygen into highly reactive superoxide radicals (⋅O^2−^​), as this potential is more negative than the required − 0.33 V. Concurrently, the photogenerated holes in the valence band (+ 1.227 V) possess a strong enough potential to directly oxidize the dye molecules. Therefore, the efficient degradation of the organic dyes is driven by a synergistic mechanism involving both the generation of superoxide radicals by electrons and the direct oxidation of the dyes by holes^59^.

**Fig. 10:**
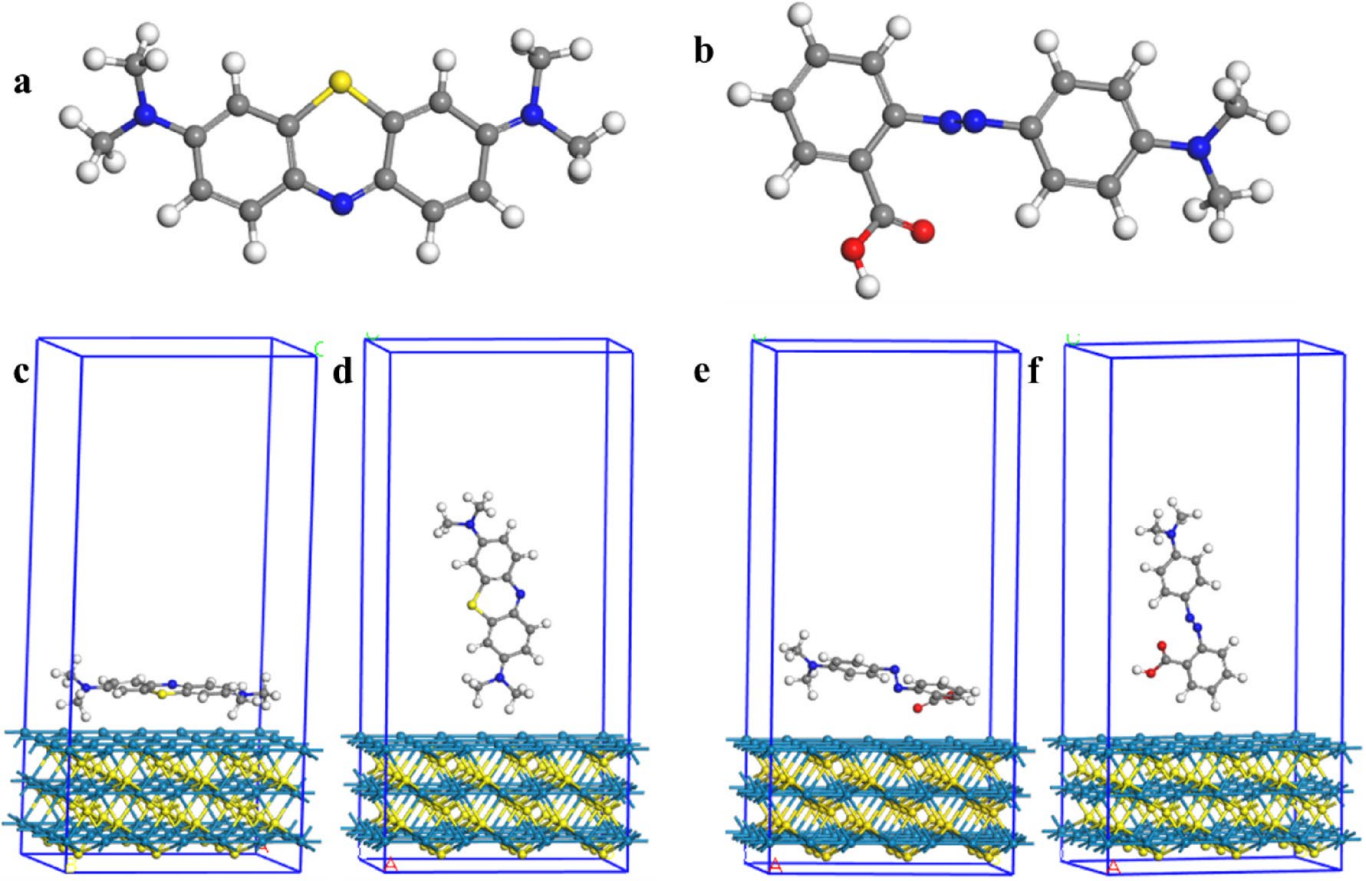
Optimized geometries of (**a**) MB, (**b**) MR, (**c**) MB adsorbed horizontally on CoS(100), (**d**) MB adsorbed vertically on CoS(100), (**e**) MR adsorbed horizontally on CoS(100), and (**f**) MR adsorbed vertically on CoS(100). Color representation: Hydrogen (white), Oxygen (red), Nitrogen (blue), Sulfur (yellow), Carbon (grey), and Cobalt (azure).

To gain deeper insights into the adsorption of MB and MR dyes on CoS, density functional theory (DFT) calculations were performed. The geometries of MB and MR were optimized, as shown in Fig. 10a and b, respectively. The orientation of both methylene blue dye (MB) and methyl red dye (MR) on the CoS surface was investigated. The horizontal orientation of MB is shown in Fig. 10c, while the vertical orientation is displayed in Fig. 10d. Similarly, the horizontal orientation of MR is presented in Fig. 10e, and the vertical orientation in Fig. 10f. The adsorption energy of MB on CoS in the vertical orientation was more favorable than in the horizontal one (−3.1 eV vs. −1.5 eV). The same behavior was observed for MR adsorption, with the vertical orientation being more favorable than the horizontal one (−2.8 eV vs. −1.2 eV). The calculated adsorption energy values indicate that MB and MR molecules are strongly adsorbed on the CoS surface, with MB exhibiting stronger adsorption than MR.

The typical distances between the surface plane of CoS and the hydrogen atoms of the MB molecule (HMB) are 2.31 Å and 2.42 Å, respectively, according to Co-HMB. Similarly, the average distances between the surface plane of CoS and the hydrogen atoms of the MR molecule (HMR) are 2.57 Å and 2.59 Å, respectively, as measured by Co-HMR. Monodentate, bidentate chelating, and bidentate bridging were proposed as three common coordinating strategies by Pastore et al.^60^. Our research revealed that MB and MR molecules are adsorbed on the (100) surface of CoS in a bidentate chelating configuration, which, as noted by various authors, results in enhanced stability of adsorption accompanied by a higher exothermic adsorption energy^61^. Consequently, estimating the adsorption energy provides information into the degree of adhesion and the configuration of molecule–surface interactions. An elevated adsorption energy signifies enhanced dye retention on the surface, which is advantageous for following photosensitive operations. These theoretical findings are consistent with the experimental observations from the photodegradation studies. Although adsorption kinetics and isotherm models were not performed experimentally, the significant dye removal efficiency observed under visible-light irradiation qualitatively supports the strong adsorption predicted by the DFT calculations. The higher adsorption energy of MB compared to MR, as indicated by the computational analysis, correlates with the experimentally observed superior degradation performance of MB. This coherence between the theoretical adsorption energies and the experimental photodegradation trends serves as an indirect validation of the computational predictions.

## Conclusion

This study demonstrates the potential of cobalt sulfide (CoS) as an efficient and cost-effective photocatalyst for visible-light-driven degradation of organic pollutants. The synthesized CoS exhibited a mesoporous structure with a BET surface area of 33.6 m²·g⁻¹ and a narrow band gap of 1.6 eV, enabling strong visible-light absorption. Under optimized conditions, CoS achieved photodegradation efficiencies of 97.7% for methylene blue (MB) and 75.3% for methyl red (MR) within 90 min, with corresponding pseudo-first-order rate constants of 0.03 min⁻¹ and 0.01 min⁻¹, respectively. Density functional theory (DFT) studies further revealed stronger adsorption energy and enhanced interaction of MB compared to MR, providing mechanistic insights into its superior performance. Additionally, CoS demonstrated good stability during repeated cycles, underscoring its practical viability for wastewater remediation. These findings highlight the dual strength of experimental and theoretical approaches in advancing next-generation photocatalysts and open pathways for scaling up CoS-based systems for real-world water treatment applications.

## Data Availability

The datasets used and/or analysed during the current study available from the corresponding author on reasonable request.
